# De Novo Transcriptome Sequencing and Analysis of the Cereal Cyst Nematode, *Heterodera avenae*

**DOI:** 10.1371/journal.pone.0096311

**Published:** 2014-05-06

**Authors:** Mukesh Kumar, Nagavara Prasad Gantasala, Tanmoy Roychowdhury, Prasoon Kumar Thakur, Prakash Banakar, Rohit N. Shukla, Michael G. K. Jones, Uma Rao

**Affiliations:** 1 Division of Nematology, Indian Agricultural Research Institute, New Delhi, India; 2 School of Computational & Integrative Sciences, Jawaharlal Nehru University, New Delhi, India; 3 School of Veterinary and Life Sciences, Murdoch University, Perth, Australia; 4 Bionivid Technology [P] Ltd, Bangalore, India; INRA, France

## Abstract

The cereal cyst nematode (CCN, *Heterodera avenae*) is a major pest of wheat (*Triticum spp*) that reduces crop yields in many countries. Cyst nematodes are obligate sedentary endoparasites that reproduce by amphimixis. Here, we report the first transcriptome analysis of two stages of *H. avenae*. After sequencing extracted RNA from pre parasitic infective juvenile and adult stages of the life cycle, 131 million Illumina high quality paired end reads were obtained which generated 27,765 contigs with N50 of 1,028 base pairs, of which 10,452 were annotated. Comparative analyses were undertaken to evaluate *H. avenae* sequences with those of other plant, animal and free living nematodes to identify differences in expressed genes. There were 4,431 transcripts common to *H. avenae* and the free living nematode *Caenorhabditis elegans*, and 9,462 in common with more closely related potato cyst nematode, *Globodera pallida*. Annotation of *H. avenae* carbohydrate active enzymes (CAZy) revealed fewer glycoside hydrolases (GHs) but more glycosyl transferases (GTs) and carbohydrate esterases (CEs) when compared to *M. incognita*. 1,280 transcripts were found to have secretory signature, presence of signal peptide and absence of transmembrane. In a comparison of genes expressed in the pre-parasitic juvenile and feeding female stages, expression levels of 30 genes with high RPKM (reads per base per kilo million) value, were analysed by qRT-PCR which confirmed the observed differences in their levels of expression levels. In addition, we have also developed a user-friendly resource, *Heterodera* transcriptome database (HATdb) for public access of the data generated in this study. The new data provided on the transcriptome of *H. avenae* adds to the genetic resources available to study plant parasitic nematodes and provides an opportunity to seek new effectors that are specifically involved in the *H. avenae*-cereal host interaction.

## Introduction

The extent of crop losses caused by plant parasitic nematodes is substantial and contributes to significant reductions in crop yields resulting in an annual yield losses of about US$157 billion [1]. The cereal cyst nematode (CCN), *Heterodera avenae* (Wollenweber, 1924), is one of the three economically important cyst nematode species that attack wheat and barley crops in many cereal growing regions of the world [2]. Although agronomic management practices are often deployed to manage plant nematodes, control with chemical nematicides is also used for high value crops. Because chemical nematicides are toxic and persistent, they are a human health risk and most are being phased out [3]. There is therefore an urgent need to find new gene targets which can be used to develop novel and environmentally friendly nematode control methods [1], [4].

Cyst nematodes are obligate sedentary endoparasites that reproduce mainly by amphimixis. After infection of host plants, they develop a close interaction with and feed from a group of interconnected syncytial cells of their host plants. The life cycle starts with an egg present inside an encysted female. The first stage larva (J1) develops within the egg and second stage larva (J2) hatches in response to low soil temperatures (5–15°C) [5]. J2s may invade main or lateral roots in the zone of elongation and migrate intracellularly towards the vascular cylinder [6]. Each J2 selects a single cell that becomes the initial feeding cell (IFC). In a susceptible host, the cells next to the IFC expand and become interconnected after local dissolution of cell walls at pit fields, resulting in the formation of a multinucleate syncytium with characteristics of transfer cells [7]. J2s feed from associated syncytia and undergo three molts, during which they develop either into a mobile male or become a sedentary endoparasitic female that continues to feed until reproduction is completed. The female then dies and forms a cyst which protects the eggs inside it. As a survival strategy not all J2s hatch simultaneously in the same season, but hatching can occur over several years with some unhatched eggs retained within the cyst [8].

Nematodes are amongst the earliest recognised parasites of plants and animals. However, plant parasites have been studied in less detail than many animal parasites and free living nematodes. Most studies have focused on the free living model nematodes, *Caenorhabditis elegans* and *C. briggsae* [9] and comparative studies with such nematodes have been useful in annotating genes and genomes of plant parasites [10]. To date, genetic resources of plant parasitic nematodes have been based mainly on EST sequences [11] and from annotated genomic sequences of the root-knot nematodes, *Meloidogyne incognita* and *M. hapla* [1], [12]. Comparatively less sequence information is available for *Heterodera* spp than for root knot nematodes, exceptions being *H. schachtii* and *H. glycines* [13], [14]. In addition, the potato cyst nematode *Globodera pallida* genome has been published [15]. Recently, transcriptomes of two migratory endoparasitic root lesion nematodes, *Pratylenchus coffeae* and *P. thornei* have also been published [16], [17].

Next generation sequencing (NGS) has greatly increased our ability to analyse transcriptomes and genomes of many organisms in a cost-effective manner [18]. In this study, using Illumina short reads, we have sequenced, assembled and annotated transcriptomes of two life stages of *H. avenae*, for which very little genomic data was available previously. Data is presented on gene transcripts expressed in two developmental stages of *H. avenae*, and transcript levels have been compared between these developmental stages. The data also provide information on potential targets for control of *H. avenae*. Overall, the *H. avenae* transcriptome provides significant new information on expressed genes, which adds to overall understanding of its parasitic abilities, and which could be used to develop novel management strategies.

## Methods

### Nematode collection and multiplication on wheat plants

Cysts of *H. avenae* were collected from infested wheat roots growing in the plots of the Indian Agricultural Research Institute, New Delhi, India. Genetic homogeneity of the population was determined by PCR-RFLP and sequencing of ITS (Internal Transcribed Spacer) regions of rDNA (ribosomal DNA). A genetically homogenous population was then multiplied on wheat plants grown in pots kept in a greenhouse [19]. Newly formed cysts were collected manually from the wheat roots using a dissecting microscope and kept for hatching. Hatched J2s were collected and later used for RNA extraction. Likewise, hatched J2s were also used to infect wheat roots and feeding females (FF) that developed and protruded from roots 6–8 weeks after infection were collected and frozen (−80°C) for RNA extraction.

### RNA extraction, cDNA synthesis, library preparation and sequencing

Total RNA was extracted from the frozen J2s and FFs using TRIzol (Invitrogen Life Technologies) according to the manufacturer's instructions. Extracted RNA was assessed for quality and quantity using an Agilent 2100 Bioanalyzer (Agilent Technologies). RNA with an RNA integrity number (RIN) of 8.0 was used for mRNA purification (Figure S1). mRNA was purified from 1 µg of intact total RNA using oligodT beads (TruSeq RNA Sample Preparation Kit, Illumina). The purified mRNA was fragmented at elevated temperature (90°C) in the presence of divalent cations and reverse transcribed with Superscript II Reverse Transcriptase (Invitrogen Life Technologies) by priming with random hexamers. Second strand cDNA was synthesized in the presence of DNA polymerase I and RNaseH. The cDNA was cleaned using Agencourt Ampure XP SPRI beads (Beckman Coulter). Illumina adapters were ligated to the cDNA molecules after end repair and addition of an ‘A’ base followed by SPRI clean-up. The resultant cDNA library was amplified using PCR for enrichment of adapter ligated fragments, quantified using a Nanodrop spectrophotometer (Thermo Scientific) and validated for quality with a Bioanalyzer (Agilent Technologies). It was then sequenced using the Illumina GAIIx platform at Genotypic Technology Private Limited, Bangalore, India. The sequence data generated has been deposited in the Array Express database for public access (Array Express accession: E-MTAB-2221, ENA accession: ERP004648).

### De novo transcriptome assembly and analysis

High quality filtered paired and orphan sequence reads (Phred Score >20) obtained from J2s and FFs were merged and used as an input for assembly of the transcriptome [20]. The Velvet-Oases pipeline was used for *de novo* transcriptome assembly (Velvet assembly is based on de Bruijn graph [21]). Assemblies with different k-mer sizes were used to assess the optimal assembly range. The Oases module was used to generate unique transcripts from the merged assembly. The assembled transcripts with sequence length longer than 100 bp were then considered for transcript annotation and quantitation. Assembly was validated by aligning raw reads back to transcripts [20]. Reads were aligned using Bowtie [22] with default parameters.

### Annotation and quantification of the transcriptome

Annotation for all the unique transcripts (>200 bp) was done using BLAST [23], homology search against Uniprot [24], the National Center for Biotechnology Information (NCBI)-NR Protein database [25] and NEMABASE4 (http://www.nematodes.org/nembase4/). In addition, BLASTX was performed to identify homologues in other databases including Refseq, Swissprot [26], European Molecular Biology Laboratory [27] (EMBL), DNA Databank of Japan [28] (DDBJ), Protein Information Resource [29] (PIR) and Protein Data Bank [30] (RCSB). Nematode orthologues were identified from other completely sequenced genomes by the reciprocal blast method [23]. To study gene orthologues across nematode species, we used the predicted protein sets from seven genomes/transcriptomes available in the public domain (Wormbase, NCBI and Sanger) *viz.*, *C. elegans*, *M. hapla*, *M. incognita*, *Pristionchus pacificus, G. pallida, P. thornei* and *Ascaris suum*. BLASTP hits with e-value scores ≤1e-5 and query coverage above 60% were considered as annotated homologous proteins and Python script was used for filtering reciprocal best hits. The *Heterodera avenae* transcriptome database (HATDB) is a freely available resource. The database was developed using platform independent and open-source software's MySQL server, PHP, HTML and CSS.

KEGG homologues were identified using the KEGG Automated Annotation Server (KAAS) with default parameters. The resulting BLAST hits were processed by BLAST2GO software [31] to retrieve associated GO terms describing biological processes, molecular functions and cellular components. BLAST2GO uses specific gene identifiers and accession numbers to generate GO annotations as well as corresponding enzyme commission numbers (EC) for sequences with an e-value≤1e-5. Homologues of the *C. elegans* RNAi pathway and phenotype genes were also identified in the *H. avenae* transcriptome by performing TBLASTX with e-value≤1e-5. The CAZymes database was searched to find enzymes involved in carbohydrate metabolism by the CAZymes Analysis Toolkit (CAT) [32] with an e-value threshold of 0.01, a bit score threshold of 55 and rule support level of 40. For further confirmation, BLAST and Enzyme Commission results were incorporated. *H. avenae* transcripts were studied for the presence of parasitic effectors identified in other *Heterodera* spp and reported in the published literature. Peptides potentially secreted were identified using SignalP [33] and those with trans-membrane motifs were removed using TMHMM [34]. SecretomeP 1.0 was used to identify non-classical secretion with default parameters [35]. Repeat elements were identified using Repeat Masker version 3.3.0 [36]. Repeat Masker was installed with RMBlast version 2.2.23+ and Tandem Repeats Finder 4.04 [37]. The transcripts were used as queries against Repbase version 20120418. Default parameters were used with the specification of species as “Nematoda”. Short Sequence Repeats (SSRs) with default parameters were identified with the help of Perl script program MISA (MIcroSAtellite; http://pgrc.ipk-gatersleben.de/misa). Stringent detection criteria were implemented to obtain perfect SSR motifs. Threshold search criteria in MISA script were considered to be appropriate if repeats of mono-nucleotides were present 12 times, di-nucleotides repeated more than 8 times and tri-, tetra-, penta- and hexa-nucleotide repeated 5 times. Expression levels of all the transcripts in the individual libraries (J2 and FF) were assessed by mapping high quality (HQ) filtered reads using BOWTIE2 [38]. Mapped reads were further normalized using RPKM method.

### Putative biological significance analysis and Biological Analysis Network (BAN) modeling

Based on the results of transcript quantitiaion, GO Elite tool [39] was used to identify statistically significant regulated GO categories and KEGG pathways with a stringent cut off level of False Discovery Rate (FDR)<0.05 for regulated transcripts. Transcripts with statistically significant homologies to GO terms and KEGG pathway genes identified by GO Elite tool were used as input for Biological Analysis Network (BAN). Cytoscape v8.1 was used to cluster the genes and processes using edge weighted force directed (Bio-layout). A network analyser plugin was used to identify enriched biological categories and regulated genes. Enriched nodes and edges were network modeled using a hierarchical layout algorithm.

### Quantitative Real-Time PCR analysis

Total RNA was extracted from J2s and the FFs of *H. avenae* using a MN NucleoSpin RNA extraction kit (Macherey-Nagel). The quality and concentration of each RNA sample was determined using RNA 6000 Nanokit and a Bioanalyzer (Aglient 2100). Only those samples having RIN values more than 7.0 were used for analysis. cDNA was synthesized from 300 *ng* of each of the RNA samples using a cDNA synthesis Kit (Superscript VILO, Invitrogen). Quantitative real-time PCR (qRT-PCR) was performed using SYBR Green I as reporter dye in a Realplex^2^ thermal cycler (Eppendorf). A master mix for each PCR reaction was prepared with SYBR Green I, ROX passive reference, stabilizers and PCR Core Reagents (Eurogentec). 1.5 *ng* of cDNA and 750 *nM* of each specific primer was added (the primers used in qRT-PCR are listed in Table S1). The following amplification program was used: 95°C 5 min, 40 cycles at 95°C 15 seconds followed by 60°C for 1 min. The *18S rRNA* gene was used as an internal control. The average ct mean values of two biological replicates were taken for calculating the fold change (2^−ΔΔCT^) [40]. Fold change values of highly expressed genes in female were calculated by comparing with J2s and vice versa. There were two biological and three technical replicates for each sample.

## Results

### Transcriptome sequencing and assembly

mRNA sequencing using the Illumina GAIIx platform yielded a total of 144 million reads of 100 base read lengths from the J2s and FF transcriptomes. Velvet assembly was done with various k-mer ranges and optimal assembly was attained at k-mer 83. Oases tool was used to identify non-overlapping isoforms/splice variants with an insert length of 150 bp and standard deviation of 50 bp. The final assembly resulted in 27,765 transcripts with an average length of 680 bp and N50 of 1,028 bp (Table 1) [(Raw data statistics) (Table 2)]. Duplicates were then removed by merging transcript assemblies from k-mer-79 to 83. The N50 value was improved after running Oases (1,028): 24,888 contigs (86%) were longer than 200 bp, with 22% of the total contigs between 200–300 bp, 43% between 300 and 900 bp, and 21% longer than 900 bp (Figure S2).

**Table 1:** Details of raw data and quality control used for assembly of the *H. avenae* transcriptome.

Parameters	Juvenile	Feeding Female
**Total Number of Reads**	92228826	74250262
**HQ Paired End Reads**	65264470	66168842
**Orphan Reads**	10210925	3224372
**Low Quality Reads**	16753431	4857048

**Table 2:** Assembly statistics of *H. avenae* transcriptome generated by Velvet and Oases.

Assembly statistics of *H. avenae* transcriptome generated by Velvet				
**Kmer length**	71	75	79	83
**Assembled reads**	83346228	80457236	79060568	78113600
**Total reads**	144868609	144868609	144868609	144868609
**Percentage assembled**	57.5323	55.5381	54.574	53.9203
**Total No. of Contigs**	49587	42782	36482	30201
**Total assembly length (bp)**	22863966	21458075	19925639	18289226
**Max contig length (bp)**	10420	8935	9073	8997
**Min contig length (bp)**	141	149	157	165
**Average sequence length (bp)**	461.088	501.568	546.177	605.583
**N50 (bp)**	596	666	748	859

Assembly statistics of *H. avenae* transcriptome generated by Oases	
Parameters	Readings
**Transcripts (Unique transcripts)**	27765
**Total Assembly length (Mb)**	18.93
**Min transcript length (bp)**	101
**Max transcript length (bp)**	9075
**Average sequence length (bp)**	682
**N50 (bp)**	1028

### Characterisation of *H. avenae* transcripts

**Figure 1:**
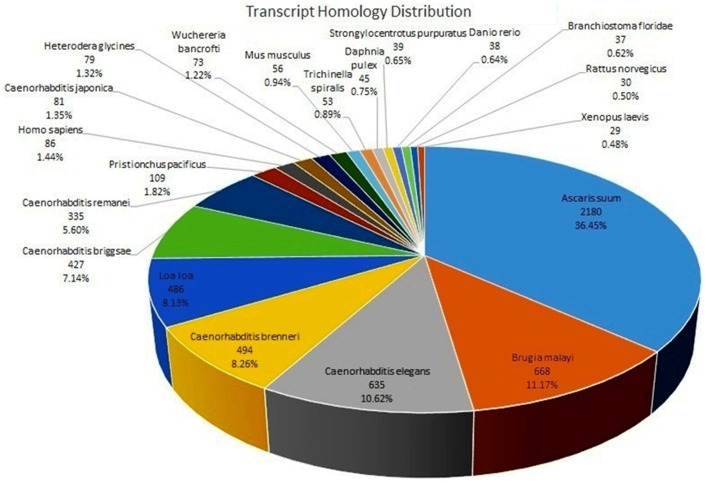
Distribution of the top 20 species with most homologues to *H. avenae*. The distribution was calculated using best BLASTX hit.

*H. avenae* transcripts were annotated by comparing the sequences obtained against non-redundant protein sequences available in GenBank and Refseq, Swissprot, EMBL, DDBJ, PIR and RCSB using BLASTX. From the analysis, 10,452 (37.64%) transcripts out of 27,765 were annotated based on the BLASTX score using an e value cutoff of 1e-5 and with minimum query coverage of 50% (Table S2). *H. avenae* transcripts showed significant hits primarily to animal parasitic nematodes (3,387) followed by free living nematodes (2,081) (Figure 1). Some genes known to be important in plant parasitic nematodes such as FLPs (FMRFamide-like peptides), MSPs (Major Sperm Proteins), *Expansin*, NLPs (Neuropeptide-like proteins) and *cysteine proteases* were identified amongst the transcripts (Table S2).

**Figure 2:**
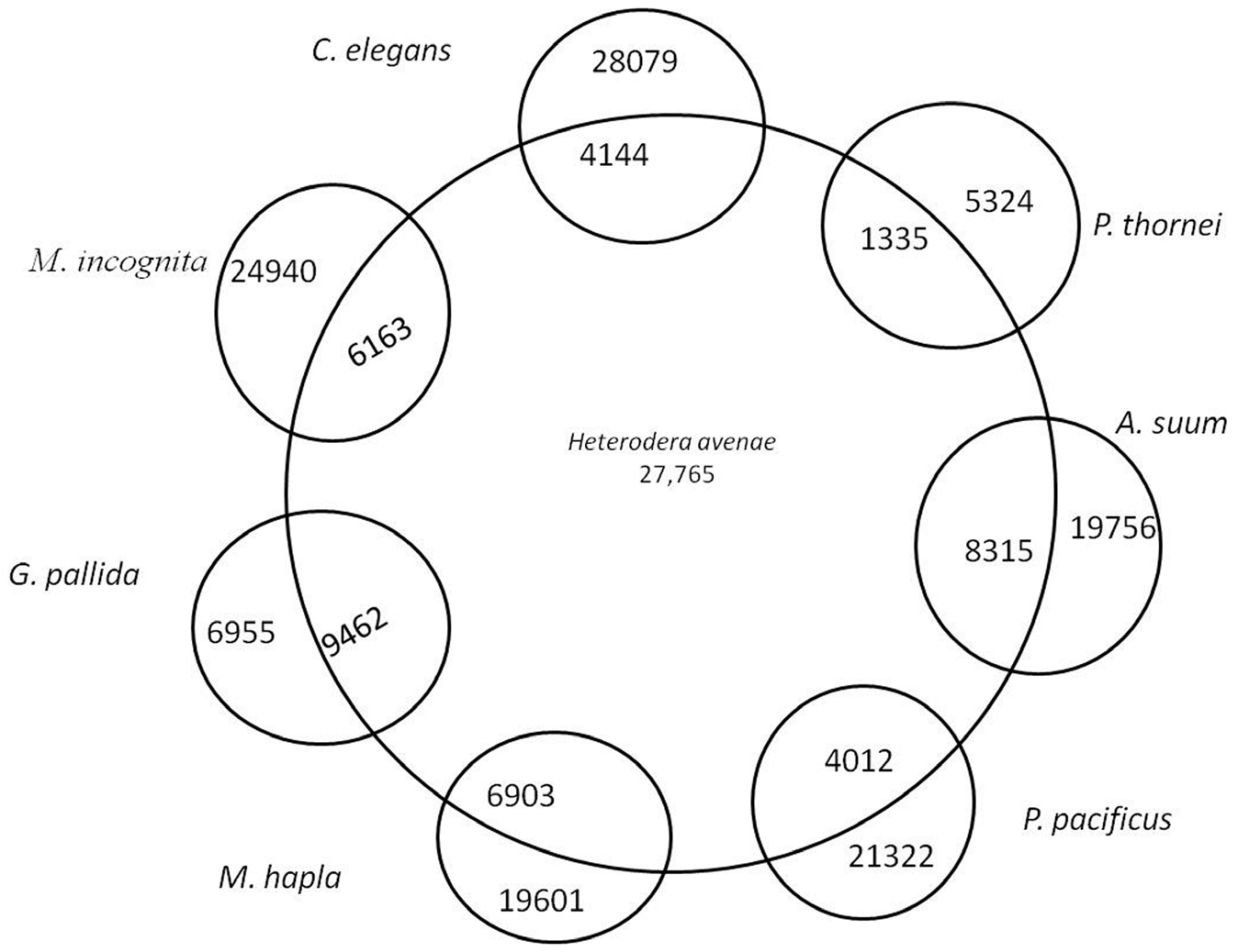
*H. avenae* orthologues present in selected completely sequenced genomes of free living (*C. elegans, Pristionchus pacificus*), animal parasitic (*A. suum*) and plant parasitic (*M. incognita, M. hapla, G. pallida*, and *Pratylenchus thornei*) nematodes. The numbers represent the common sequences.

The *H. avenae* transcripts were compared with completely sequenced genomes of nematodes using the reciprocal blast method to assign putative orthologues. *H. avenae* sequences shared 6,163 orthologues with *M. incognita*, 6,903 with *M. hapla* and 1,335 with *P. thornei*. It also shared 4,144 orthologues with the free living nematode, *C. elegans*, and 8,315 sequences with the animal parasitic nematode, *Ascaris suum* (Figure 2). The species with the most shared sequences was *G. pallida* with 9,462 common transcripts.

Transcripts present in other nematodes in the form of expressed sequence tags (ESTs) were also identified in *H. avenae* using NEMABASE4 that houses EST data of 62 nematode species [10]. To identify the extent of this similarity, *H. avenae* transcripts were compared with NEMABASE4 annotated EST data sets of all the nematodes using BLASTN (e -value≤1e-5) (Table S3a, b) [11]. The analysis revealed a total of 4,836 significant hits of which 96.42% of the transcripts matched homologues of Heteroderidae according to best BLASTN hits without many hits with other nematodes. This may be due to nucleotide level variation among different group of nematodes [41]. BLAST searches with EST (Table S4) data could annotate 1,839 transcripts which did not show homologues in NR database (Table S3b).

The majority of *C. elegans* genes have been surveyed for knockout phenotypes using RNAi, where matching mRNA is degraded by the introduction of sequence-specific double-stranded RNA (dsRNA) [42]. A comparative analysis was done between *H. avenae* contigs and *C. elegans* genes to identity homologues of genes with RNAi phenotypes in *C. elegans*: 4,147 contigs of *H. avenae* were found to be homologous to 2,543 genes of *C. elegans* with at least one reported RNAi phenotype (Table S5). Primary sequence similarity based searches were then undertaken to identify putative homologous RNAi pathway genes of *C. elegans* in *H. avenae*, and 30 RNAi pathway gene homologues were found in the *H. avenae* transcriptome (Table 3).

**Table 3:** Comparative analysis of putative RNAi pathway genes in *H. avenae*, *C. elegans* and *M. incognita*.

*C. elegans*	*H. avenae*	*M. incognita*
**Dicer complex**		
**dcr-1**	+	+
**drh-1**	+	+
**drh-3**	+	+
**xpo-1**	+	+
**xpo-2**	+	+
**drsh-1**	+	+
**rde-4**	-	+
**rde-5**	-	+
**RISC complex**		
**alg-1**	+	+
**alg-2**	-	+
**alg-4**	+	+
**ain-1**	+	+
**vig-1**	-	+
**tsn-1**	+	+
**RO6C7.1**	-	+
**CO4F12.1**	-	+
**F58G1.1**	-	+
**T22H9.3**	-	+
**csr-1**	+	+
**sago-1**	+	-
**ppw-1**	-	-
**ppw-2**	+	-
**nrde-3**	+	-
**RdRp amplification complex**		
**ego-1**	+	+
**rrf-1**	+	+
**rrf-3**		
**Systemic RNAi (spreading)**		
**rsd-2**	-	-
**rsd-3**	+	+
**rsd-6**	-	-
**sid-1**	-	-
**sid-2**	-	-
**Required for RNAi**		
**zfp-1**	+	+
**smg-2**	+	+
**smg-6**	-	-
**mes-8**	-	+
**mes-6**	-	-
**mes-2**	+	+
**mut-2**	+	-
**mut-7**	+	-
**gfl-1**	+	-
**cid-1**	+	+
**ekl-4**	-	+
**ekl-1**	+	+
**rha-1**	+	+
**gfl-1**	+	-
**RNAi inhibitors**		
**eri-1**	+	+
**eri-7**	+	-
**xrn-2**	+	+

### *Heterodera avenae* transcriptome database (HATdb)

The data in the HATDB can be easily accessed. HATDB is available on the URL http://insilico.iari.res.in. Users can query the database by providing various options for search of nucleotide and protein like GO (Biological component, Molecular activity and Molecular process) Pfam id. Transcript Identifier. The sequence-based search has been employed using Viro-BLAST [23], [43] search which provides the user friendly option of different BLAST algorithms to search and download nucleotide or protein sequence (s) against *Heterodera avenae* transcriptome sequence data reported in this study. In addition, the links have also been provided to download the whole assembled transcripts and predicted protein.

### Putative functional classification using Gene Ontology and KEGG pathway analysis

**Figure 3:**
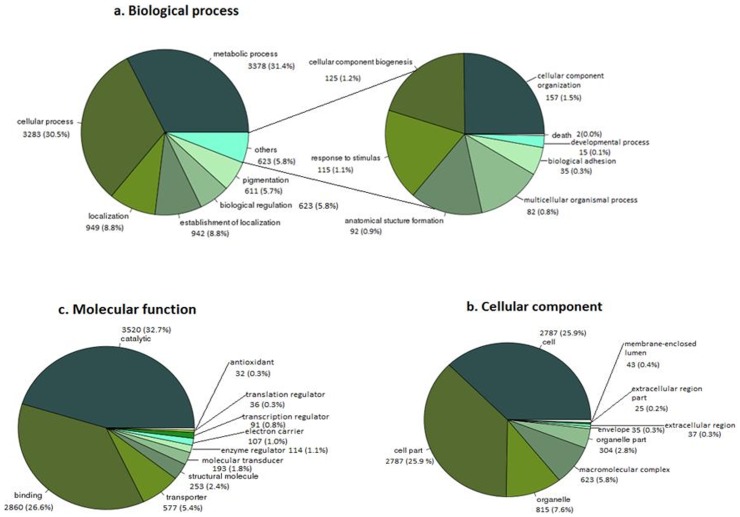
Functional classification of *H. avenae* transcripts using Gene ontology (GO) terms, a. Biological process, b. Molecular function, c. Cellular component.

GO terms were assigned to 10,751 transcripts for further functional characterisation (Figure 3). In this analysis, the predominant transcripts identified as genes involved in catalytic activity constituted 32.7% of the transcripts, of which 10.2% were involved in hydrolytic activity and 0.8% in lyase activity, and 26.6% were indentified with binding activity, of which 0.3% were carbohydrate binding. Amongst those categorized under biological processes, transcripts for genes involved in metabolic processes (31.4%) and cellular processes (30.5%) were well represented. Results also indicated that 403 transcripts were homologous to genes involved in cellular communications (Figure 3) [44]. Neuropeptides that are responsible for neuro-muscular activity comprised 0.3% of the transcripts [45]. In addition, 15 transcripts appeared to be involved in cell cycle regulation (Figure 3) [46]. Metabolic pathways were assigned to *H. avenae* contigs using the Kyoto Encyclopedia of Genes and Genomes database for determining their biochemical functions [47]. Many of the transcripts matched genes in KEGG pathways; metabolic pathways (479), biosynthesis of secondary metabolites (144), oxidative phosphorylation (66), biosynthesis of phenylpropanoids (1) and ubiquitin mediated proteolysis (60). There were 20 KEGG Orthology (KO) mapped to the lipid biosynthesis pathway (Table S6a, b).

### Transcriptome quantitation and enrichment of significant biological categories

**Figure 4:**
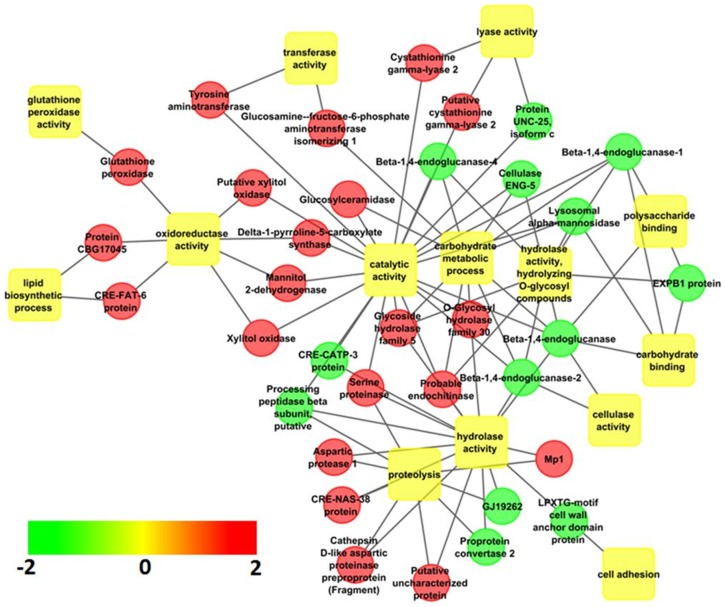
Hierarchical layout of significantly enriched biological processes and key regulatory genes in *H. avenae*.

*In silico* quantitation of transcripts was done by mapping the reads from individual libraries (J2 and FF) using BOWTIE2 [38], which provided an estimate of transcript abundance. Regulated genes were subjected to GO term enrichment analysis using GOElite V1.2.5 (http://www.genmapp.org/go_elite/). Analysis revealed 64 biological categories under GO terms and pathways significantly enriched at the stringent cut off level of False Discovery Rate (FDR) <0.05 (Table 4). To identify the major functional themes, enriched GO terms were organised into a functional network with 34 nodes and 14 edges using enrichment map plugin in Cytoscape v 8.1 (Figure 4). Force directed layout visualisation of the enrichment map showing FDR-based sub clustering revealed four functional clusters including; a) carbohydrate binding b) proteolysis c) motor activity d) hydrolase activity, hydrolyzing O-glycosyl compounds (Figure 4). Visualisation of sub-clusters using hierarchical layout suggested that hydrolase activity was up-regulated in J2s whereas binding activity was up-regulated in FFs.

**Table 4:** Statistically significant regulated Gene Ontology categories and pathways in *H. avenae*.

Category	Term	Highly Expressed*	Z Score	p Value	q Value
**Cellular component**	integral to membrane (GO:0016021)	36	1.87	0.23	1.00
**Biological process**	proteolysis (GO:0006508)	11	1.84	0.16	1.00
**Molecular function**	calcium ion binding (GO:0005509)	10	1.68	0.20	1.00
**Biological process**	G-protein coupled receptor protein signaling pathway (GO:0007186)	7	2.15	0.08	1.00
**Molecular function**	hydrolase activity, hydrolyzing O-glycosyl compounds (GO:0004553)	11	7.34	0.00	0.11
**Molecular function**	motor activity (GO:0003774)	4	2.88	0.04	1.00
**Molecular function**	carbohydrate binding (GO:0030246)	6	5.63	0.00	0.17
**Cellular component**	collagen (GO:0005581)	6	7.03	0.00	0.11
**Biological process**	fatty acid biosynthetic process (GO:0006633)	2	1.95	0.13	1.00
**Biological process**	response to oxidative stress (GO:0006979)	4	4.68	0.00	0.08
**Molecular function**	peroxidase activity (GO:0004601)	3	3.55	0.02	0.76
**KEGG**	ABC transporters:KEGG-ko02010	3	5.52	0.00	0.24
**KEGG**	Butanoate metabolism:KEGG-ko00650	3	3.77	0.02	1.00

*Based on transcript quantitiation and RPKM method.

### Validation of putative differentially expressed genes by qRT-PCR analysis

qRT-PCR was performed to identify some differentially expressed transcripts by taking cue from transcript quantitation (Table S2). Thirty genes were selected for further study based on statistical significance, annotation and presence in at least one developmental stage (J2 or FF) of *H. avenae*. Amongst them, 15 genes were expressed highly in the FF stage *viz*., (*Spectraplakin, Ran-BPM,CAEBREN_25536, CAEBREN_07082, Receptor family ligand binding region containing protein, Chitinase-like protein, Superoxide dismutase, Oxidoreductase dhs-27, ‘Paired box’ domain containing protein, Chitin binding Peritrophin-A domain containing protein, CBN-FAT-5 protein, dorsal gland cell-specific expression protein, CRE, CBN-AQP-4, CLAVATA*) and the remaining 15 were up-regulated in the J2 stage (*Pectate lyase, Serine protease, β-1-4-endoglucanase, Annexin A8, Putative gland protein G12H04, CAEBREN_17602, CAEBREN_19301, IMG5_133010, CLF_100574, CBG10845, OsI_22746, Radixin, Dynein light chain 2, pfam02414*).

**Figure 5:**
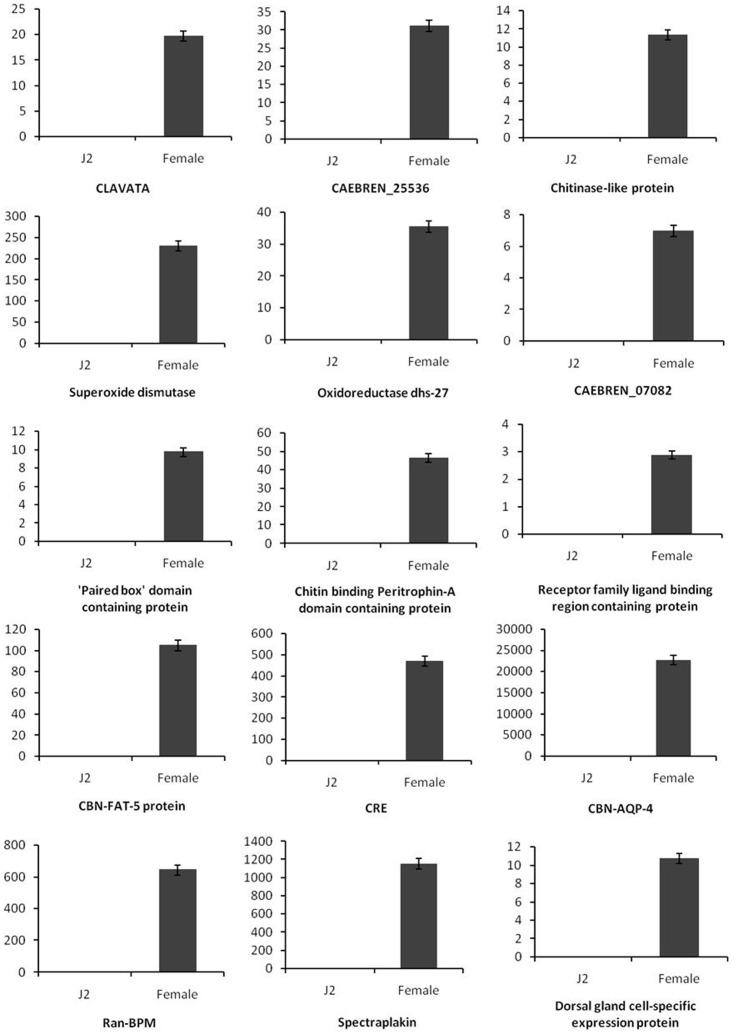
Confirmation of up regulated genes in the female stage of *H. avenae* by qRT-PCR. The Y-axis represents the log2 fold change values. Error bars show ±SD among the biological replicates. *18S rRNA* was used as an internal control gene and fold change was calculated using 2^−ΔΔCT^ method.

**Figure 6:**
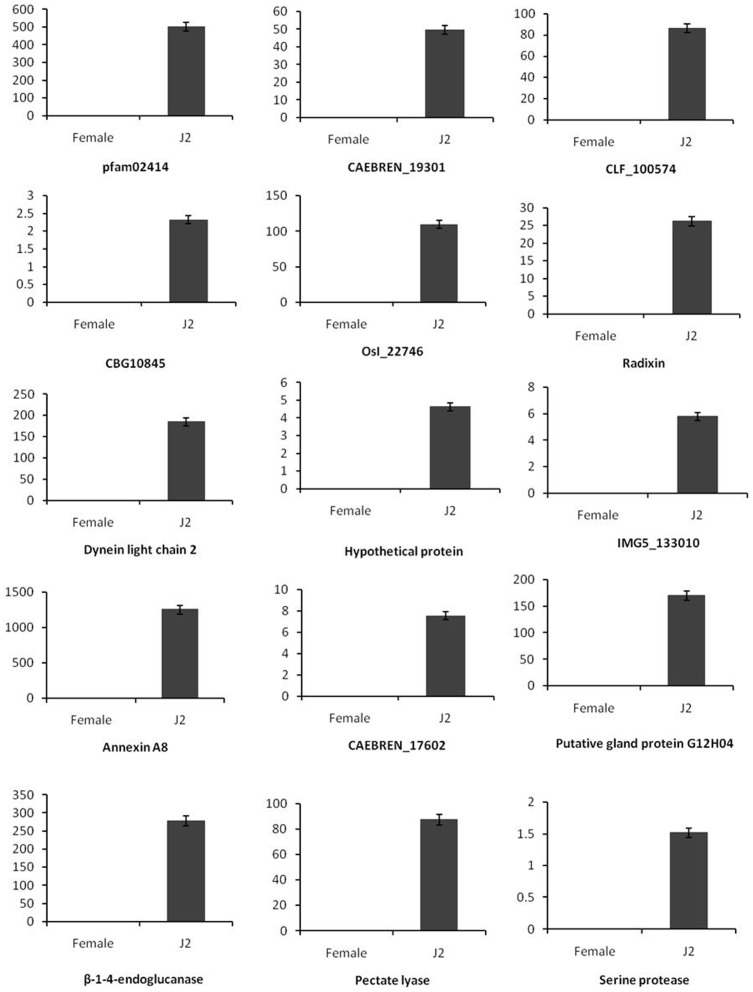
Confirmation of up regulated genes in juvenile stage of *H. avenae* by qRT-PCR. The Y-axis represents the log2 fold change values. Error bars show ±SD among the biological replicates. *18S rRNA* was used as an internal control gene and fold change was calculated by using 2^−ΔΔCT^ method.

Transcripts for *CBN-AQP-4, Spectraplakin, Ran-BPM, CRE* and *Superoxide dismutase* showed very high expression in females with the fold change values 22693,1152, 643, 471 and 230 respectively compared to J2 (Figure 5), whereas *Annexin A8, pfam02414, β-1-4-endoglucanase, Putative gland protein G12H04,OsI_22746 and Pectate lyase* showed high expression in J2 and the respective fold change values were 1251, 501, 278, 170, 109, 87 compared to female (Figure 6). qRT-PCR results were in agreement with those obtained by digital gene expression.

### Secretory peptides, CAZymes and genes involved in parasitism

Analysis to predict secreted proteins in *H. avenae* was undertaken using three approaches. Transcripts with predicted signal peptides lacking trans-membrane domains were identified by TMHMM and putative secretory sequences similar to plant host species were excluded. This analysis revealed that 1,471 of the contigs had signal peptides but lacked a trans-membrane helix (TMH). Of these, 191 showing significant similarity with the host were excluded from the analysis (Table S7). Among these homologues, 13 belonged to known effectors of *Heterodera* spp (Table 5). Homologues to some potentially interesting effectors were identified including a fatty acid retinoid binding protein RAN-BP-like protein (*Gp-rbp-1*), esophageal gland cell secretory protein (*Hg-hsp-11*), pectate lyase (*Hg-PL-2*), esophageal gland cell secretory protein 1 (*Hg-hsp-1*) and *Annexin* (*Hg-4C10*).

**Table 5:** Important genes identified in *H. avenae* based on best BLASTX hits.

Accession No.	Descriptions	Species	e-Value	%Identity	Reference
**ABV54446**	GHF5 - endo-1,4-beta-glucanase precursor	*Radopholus similis*	7.00E-23	46	[83], [84]
**ADY41344**	B 1,4-alpha-glucan-branching enzyme	*Ascaris suum*	8E-133	77	[83]
**ADL29728**	Expansin	*Heterodera glycines*	7.00E-20	75	[57]
**ADD82848**	Pectate lyase	*Heterodera avenae*	2.00E-133	97	[85]
**ACO55952**	beta-1,4-endoglucanase	*Heterodera avenae*	4E-10	81	[84]
**ACV31368**	expansin B2	*Globodera rostochiensis*	0.003	90	[57]
**ACN93668**	*Annexin* 4F01	*Heterodera schachtii*	5.00E-07	58	[86]
**Q9NFS0**	Putative hypodermis secreted protein	*Globodera rostochiensis*	3E-57	71	[87]
**AAK60209**	venom allergen-like protein-1	*Heterodera glycines*	2.00E-09	83	[88]
**NP_001123113**	Cysteine Protease related family member (cpr-6)	*Caenorhabditis elegans*	2.00E-08	77	[89]
**CAD38523**	Glutathione peroxidase	*Heterodera glycines*	0.0000004	65	[90]
**ACO35733**	Ran-Binding protein (RBP-1)	*Globodera pallida*	7E-11	43	[91]
**A4Q9L4**	Transthyretin-like protein 4	*Radopholus similis*	6.00E-69	79	[92]
**Q9BJ52**	Putative zinc finger protein	*Heterodera glycines*	6E-38	81	Unpublished
**Q86DU4**	Ubiquitin extension	*Heterodera schachtii*	3.00E-14	59	[93]
**P53021**	(Major sperm protein-1) MSP-1	*Globodera rostochiensis*	2.00E-42	83	Unpublished
**I3VB56**	Calreticulin	*Radopholus similis*	1.00E-76	91	[94]
**Q93142**	FAR-1	*Brugia malayi*	9e-17	35	[95]
**I6ZQH6**	SPRYSEC-8	*Globodera rostochiensis*			[65]
**AF159590**	Hsp-11	*Heterodera glycines*	1E-29	54	[96]
**ADY48649**	Ubiquitin-conjugating enzyme E2	*Ascaris suum*	4.50E-81	43	[97]
**ADY39814**	E3 ubiquitin-protein ligase	*Ascaris suum*	8.70E-102	55	[97]
**ADY41696**	Ubiquitin carboxyl-terminal hydrolase 8	*Ascaris suum*	9.41E-113	51	[97]
**Q7YWJ0**	MSP-21	*Meloidogyne incognita*	3.00E-124	46	[98]

Transcripts with homology to genes that might be involved in the parasitic interaction were identified such as some that modify plant cell walls (CAZymes) [48]. Identification of CAZymes was done using the CAZymes Analysis Toolkit (CAT) that classified 962 transcripts in this category (Table 6, Table S8). The number of transcripts representing different CAZyme families were glycosyl hydrolases - GHs (39), glycosyl transferases - GTs (51), pectate lyases -PLs (2), carbohydrate esterases - CEs (6) and carbohydrate binding modules - CBMs (14) (Table S8). A comparative study of the abundance of CAZymes between *H. avenae* and *M. incognita* suggested that *M. incognita* possessed more genes for hydrolytic activity. In general, CBMs are the non-catalytic domains that appends to glycoside hydrolase enzymes that degrades polysacherides [49]–[51] (Table S8). Sixty-four CBM families have been reported in the CAZy database [52] out of which seven (2, 5, 13, 14, 18, 20 and 50) were found in the *H. avenae* transcriptome.

**Table 6:** *Heterodera avenae* enzymes with predicted cell wall–degrading activities, compared with those in other nematodes.

Substrate	Cellulose			Xylan	Pectin		Arabinan
** Family**	**GH5**	**GH45**	**GH27**	**GH30**	**GH28**	**PL3**	**GH43**
*** H. avenae***	16	0	5	0	0	2	2
*** M. incognita***	21	0	4	6	2	30	2
*** B. xylophilus***	0	11	0	0	0	15	0
*** C. elegans***	0	0	0	0	0	0	0

Substrate	1,3-glucan	Chitin		
** Family**	**GH16**	**GH18**	**GH19**	**GH20**
*** H. avenae***	0	0	1	1
*** M. incognita***	0	3	2	2
*** B. xylophilus***	6	9	2	7
*** C. elegans***	0	38	6	5

GH- Glycosidehydrolases,GT-Glycosyl transferases,CE-Carbohydrate esterases, PL - Polysaccharide lyases.

### Repeat elements in *H. avenae* transcripts and identification of SSRs

The transcriptome data was also used to identify repeat elements present in *H. avenae* since there is no information on genome-wide repeats for this species. The Repeat Masker program [36] was used to identify different repetitive elements among the transcripts. Approximately 3% of the total transcripts were found to be encoded by different repetitive elements (Table S9). Low complexity regions encoded most transcripts from repetitive elements. A total of 91 retroelements were found amongst transcripts, with 43 long interspersed repeat elements (LINEs), though surprisingly, there were no short interspersed repeat elements (SINEs). Among retroelements, the number of long terminal repeats (LTRs) was slightly higher (48) than non-LTR elements. Also, 28 penelope-like elements distinct from LTR and non-LTR elements were found [53]. In addition, DNA transposons of different classes, simple repeats and small RNAs were identified. For comparison, the *C. elegans* genome contains about 524 SINE elements of which 46% are encoded in the intronic regions [54].

Perl script MISA was used to identify SSRs and generated 1,422 SSRs found in 1,125 transcripts, with a frequency of one SSR per 9.33 kb of the sequence (Table S10). Tri-nucleotide SSRs represented the largest fraction (68.3%) followed by mono-nucleotide (22.6%) and di-nucleotide (6.6%) repeats. Only a few tetra- (33) and penta-nucleotide repeat (1) SSRs were identified in the *H. avenae* transcripts.

## Discussion

In this study, we have sequenced and annotated the transcriptome of two stages of *H. avenae* after deep sequencing [55]. The combined assembled contigs from RNA-Seq of J2s and FFs generated 27,765 contigs with N50 of 1,028 bp, for which BLAST searches yielded 37% (10,454) with significant homologies to previously annotated genes in standard databases. A comparison with the EST dataset of NEMABASE4 gave 1,839 clade-specific unique hits, most from the Heteroderidae that increased transcript annotation by a further 6%; with 57% of the contigs uncharacterized. Comparative analyses of contigs of the J2/FF combined transcriptome with sequence data from other classes of nematodes also revealed homologies with free living and animal parasitic nematodes, and the available *H. avenae* contigs shared 38.9% similarity with those of the potato cyst nematode, *G. pallida*. Assignment of GO terms categorised 10,751 transcripts to putative functions.

Our analysis shows that the *H. avenae* transcriptome encodes messages most similar to those thought to be involved in parasitism by *G. pallida*. To be a successful plant pathogen *H. avenae* must locate a host plant root, enter it using its mouth stylet, and migrate intracellularly from cell-to-cell before inducing the feeding site syncytium. As has been found for other plant endoparasitic nematodes, a range of cell wall degrading enzymes have been identified, possibly required to modify plant cell walls during migration, feeding or syncytium formation. Thus, the cell wall modifying CAZymes of *H. avenae* could be expected to be involved in these processes. Thirteen GH families were found in *H. avenae* transcripts: amongst them, GH5 cellulases are most common to all the PPNs except for *B. xylophilous* where GH45 is present. Two β-1,4 endoglucanases of *G. rostochiensis* and *H. glycines* are thought to be involved in J2 migration to the final feeding site [56]. Some endoparasitic nematode ‘parasitism’ genes, particularly those potentially involved in plant cell wall modification, share a high degree of similarity with genes from bacteria and fungi. This suggests that horizontal gene transfer (HGT) has occurred during evolution to parasitism, since such genes are not present in free living nematodes [56]. The results obtained from *H. avenae* are consistent with this view.

Cyst nematodes such as *H. avenae* must form multinucleate syncytial feeding sites by altering the differentiation of a group of cells which fuse together accompanied by local wall degradation. The nuclei in syncytia become endo-polyploid, and related to this we have identified 15 transcripts which may be involved in cell cycle regulation and development of endo-polyploidy. Similarly, transcripts for *Expansins*, which are cell wall loosening proteins involved in growth and cell wall disassembly and in syncytium expansion [57] are also present in *H. avenae*. Surprisingly, *Chorismate mutase*, which is thought to be involved in evading plant defenses against invading nematodes was not found in our *H. avenae* transcriptome data [1], [58]–[60].

Genes important for the interaction between the nematode and its host are likely to be secreted into host tissues or cells. To predict putative secreted proteins encoded by *H. avenae* we identified transcripts with signal peptides with no trans-membrane helices/domain. Some of the most interesting contigs with these characteristics having no matches in any free living nematodes, could be involved in the interaction of *H. avenae* with its host. Out of the 1,280 transcripts identified to be secretory, 602 transcripts had no matches. Since parasitic nematodes have to suppress different host defense responses for their survival, they may achieve these using different mechanisms. The C-type lectin found in *H. avenae* is an interesting gene that could be involved in overcoming the host defenses as reported in animal parasitic nematodes [61], [62], since survival of *H. glycines* in host plants was significantly reduced when RNAi was used to down-regulate a C-type lectin [63]. Another important gene family, SPRYSECs has also been identified in *H. avenae* [64]. Eight types of SPRY domain-containing transcripts were identified, that might help to counter host immune responses [64], [65]. Similarly, several animal parasitic nematodes secrete antioxidant enzymes that may provide protection against host-derived reactive oxygen species (ROS). Presence of such antioxidant enzymes in plant parasitic nematodes may similarly provide protection against plant defenses [66]. We have found transcripts for the enzyme *glutathione reductase* that could provide protection to *H. avenae* against ROS. Venom-allergen-like proteins (VAPs or VALs) are highly conserved proteins released by parasitic nematodes suggesting their importance (Table 5) [67]. However, attempts to knock down VAPs in animal parasitic nematodes with RNAi have not been successful, but Lozano (personal communication) used transgenic potato plants to knock down *VAP*s in *G. rostochiensis*, and this significantly reduced the infectivity of nematodes, suggesting that *VAP*s are required for successful parasitism.

Modulation of host protein turnover rate may also be important for successful parasitism. Ubiquitination has been implicated in the regulation of many processes in plants, such as innate immunity, cell death, cell cycle regulation, hormone signaling and circadian rhythms. Plant pathogens may hijack the ubiquitination system of the host to modulate cellular processes. Presence of the ubiquitination complex components in pharyngeal glands of PPNs indicates that they may also exploit the host's ubiquitination system [68], and this is consistent with the presence of of ubiquitination complex components found in *H. avenae*.

Since *H. avenae* may survive in the soil for several years, it is of interest to understand its nutritional needs and mechanism of survival [69]. Lipids are the primary source of energy in eggs of PPNs, and lipid droplets are prominent in early J2s and FFs. Interestingly, we have identified many transcripts that mapped to the lipid biosynthesis pathway (20). PPNs secrete a specific class of fatty-acid and retinol-binding (*FAR*) proteins that may interfere with lipid-based defenses by inhibiting the production of jasmonic acid [70] in the host, and a *FAR* protein homologue is present in *H. avenae*. Similarly, *Annexins* (*Hg4F01*) are phospholipid-binding proteins in nematodes with possible immunomodulatory properties. *Annexins* bind phospholipids, the main components of cell membranes, in a calcium-dependent manner. *Annexins* are expressed in the pharyngeal and the amphidial glands of plant parasitic cyst nematodes [71] (Table 5).

Genome wide RNAi phenotypic studies in *C. elegans* have provided information on genes vital for growth and development. Comparison of *H. avenae* contigs with genes of *C. elegans* lethal RNAi phenotypes gave 4,147 homologous contigs which are expressed in both J2s and FFs of *H. avenae*. These genes are involved in various molecular functions that directly affect movement, pharyngeal pumping, lipid metabolism, embryogenesis, larval development, moulting, growth, reproduction and defence response to chemicals. An example of such phenotype in PPNs is the silencing of two genes involved in movement, *pat-10* and *unc-87*, in J2s of *P. thornei* which reduced reproduction by 77–81% on carrot mini discs. The phenotypic effect after feeding dsRNA to these nematodes was abnormal behaviour including twitching, slow movement, repeated banging of the head against the body and loss of orientation [72].

Genes involved in the RNAi pathway are well characterised in many nematode species, although there is less knowledge of the pathways involved in RNAi in PPNs compared to *C. elegans*. A total of 30 genes involved in the RNAi pathway were found amongst the *H. avenae* transcripts, including a putative nuclear AGO (Argonaute) *NRDE-3* not previously identified in a PPN (Table 3) [44] and this needs to be further investigated.

Nematodes have a primitive central nervous system (CNS) and use signaling processes involving neuropeptides [45] that have a diverse role in the function and development of nervous system [73]. They not only act as neuromodulators, but also as primary transmitters in invertebrate nervous systems [73], [74]. To date 109 neuropeptide genes have been identified in *C. elegans* [73], [75], [76]. Based on their conserved motifs, these are divided into three classes: the FMRFamide-like peptide (FLP) gene family, INS genes that encode insulin-like peptides and peptides derived from neuropeptide-like protein (NLP) genes which have no sequence similarity to the other two classes [77]. In the *H. avenae* transcriptome data, we found several neuropeptide genes belonging to all the three classes. These included *flp*-2, 3, 11and 18, four *flp*-receptors, nine NLPs and several INS genes such as Insulin-like growth factor 2. The function of *flp* gene products is better understood in *C. elegans* than in PPNs [44]. Expression studies in *C. elegans* using *flp-18* reporter gene constructs revealed its expression in the specific interneurons AVA, AIY and RIG, the motor neuron RIM and pharyngeal neurons M2 and M3 [78]. *flp-18* mutants in *C. elegans* were defective in chemosensation, foraging, dauer formation and fat accumulation and also exhibited decreased oxygen consumption. *flp-18* mutants in *C. elegans* were defective in chemosensation, foraging, dauer formation and fat accumulation and also exhibited decreased oxygen consumption. Host delivered RNAi of *flp-18* significantly reduced the infection and multiplication of *M. incognita* [79]. Similar effects could be envisaged from knock down of these genes in *H. avenae*.

In addition, to confirm the *in silico* quantitation, a selection of 30 of the putatively differentially expressed genes were checked by qRT-PCR and their relative levels of expression validated. High expression of pectate lyase and *β-1, 4 endoglucanase*, *Annexin* and putative gland protein *G12H04* supported the suggestion that in the infective J2s some of these gene products may be required for host invasion, whereas genes highly expressed in FFs are more likely to be involved in other metabolic activities such as reproduction: these results are consistent with similar earlier observations for cyst nematodes [80]. Up-regulation of *CLAVATA* in FFs could indicate their longer involvement with maintaining feeding site integration [81].

About 3% of the total transcripts encoded different repetitive elements. This may be due to lack of expression of repetitive elements or difficulty in assembling short reads containing repeat elements. However, many SSRs were identified in *H. avenae*, with more tri-nucleotide SSRs than di-nucleotide SSRs, and this agrees with observations obtained from genomic sequencing of *M. incognita* [82]. The identification of SSRs may be useful for the development of large sets of markers which would facilitate linkage mapping studies and population genetics research on *H. avenae*.

In conclusion, we have undertaken the first *de novo* analysis of the transcriptomes of two life stages of *H. avenae*, and identified some genes that may be important in plant parasitism. A comparative analysis of gene expression between the two stages of the parasite provides additional information on some genes that are likely to be involved either in parasitism or nematode metabolism. These data on the *H. avenae* transcriptome should be a valuable resource for future genomic studies on these economically important plant parasitic nematodes.

## Supporting Information

Figure S1 **Quality check of RNA by Bioanalyzer (Agilent), RNA area: 150.0, RNA concentration: 112 ng/µl, rRNA Ratio [28 s/18 s]:1.4, RNA integrity number (RIN):8.**
(TIF)

Figure S2 **Sequence length distribution of** ***H. avenae*** **assembled contigs.**
(TIF)

Table S1 **Primer information and BLASTN search results for the genes used for quantitative real time PCR.**
(XLSX)

Table S2 **Complete BLAST hit table against nr GENBANK, Refseq, Swissprot, EMBL, DDBJ, PIR and RCSB databases and transcript quantitiation.**
(XLSX)

Table S3 **(A) BLASTN result against NEMABASE4 data (B) 1,839 hits did not show any homologues in NR database.**
(XLSX)

Table S4 **Complete list of species used for nematode specific BLASTN against NEMABASE4 data.**
(XLSX)

Table S5 **RNAi lethal phenotype homologues found in the** ***H. avenae*** **transcriptome.**
(XLSX)

Table S6 **(A) Summary of KEGG pathway information for *H. avenae* transcriptome (B) KEGG pathways for 3,601 transcripts of *H. avenae* transcriptome.**
(XLSX)

Table S7 **Transcripts potentially secreted by** ***H. avenae*** **.**
(XLSX)

Table S8 **Details of all the CAZymes in the** ***H. avenae*** **transcriptome with blast and enzymes commission result.**
(XLS)

Table S9 **Expressed repeat elements identified in** ***H. avenae*** **transcriptome.**
(XLSX)

Table S10 **Details of the expressed Simple Sequence Repeats identified in** ***H. avenae*** **transcripts.**
(XLSX)
