# Supplementary figures and images for: De Novo Transcriptome Sequencing and Analysis of the Cereal Cyst Nematode, Heterodera avenae
Source: PLoS One. 2014 May 6;9(5):e96311. doi: 10.1371/journal.pone.0096311 (PMC4011697; doi:10.1371/journal.pone.0096311)

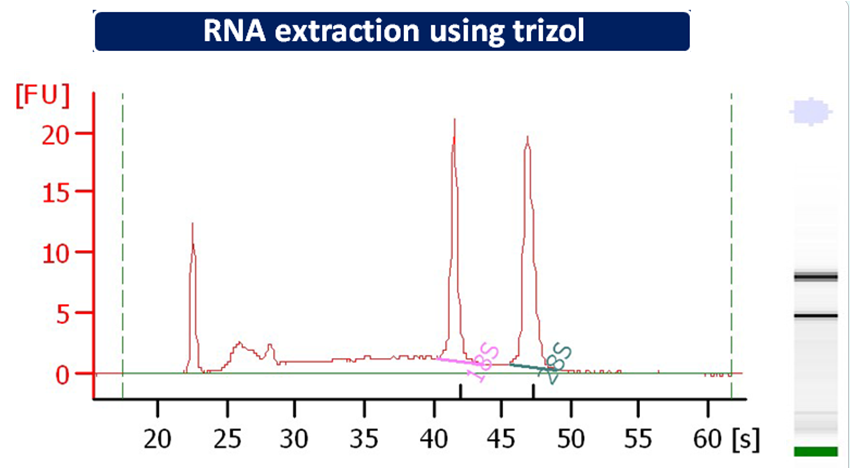

Supplement: Figure S1 — Quality check of RNA by Bioanalyzer (Agilent), RNA area: 150.0, RNA concentration: 112 ng/µl, rRNA Ratio [28 s/18 s]:1.4, RNA integrity number (RIN):8. (TIF) [file pone.0096311.s001.tif]

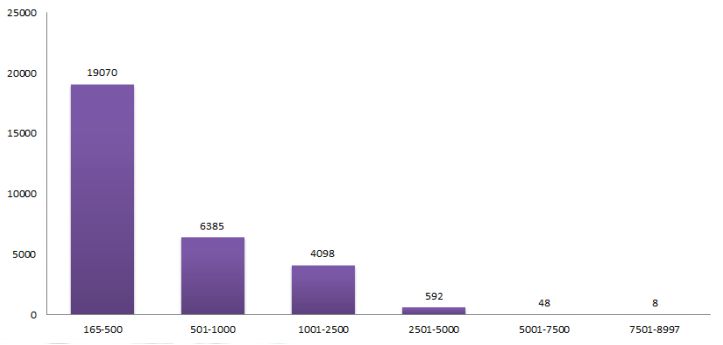

Supplement: Figure S2 — Sequence length distribution of H. avenae assembled contigs. (TIF) [file pone.0096311.s002.tif]
